# Gallbladder adenocarcinoma with human chorionic gonadotropin: a case report and review of literature

**DOI:** 10.1186/1746-1596-5-46

**Published:** 2010-07-02

**Authors:** Shinkichi Sato, Masanori Ishii, Takeaki Fujihira, Eisuke Ito, Yasuo Ohtani

**Affiliations:** 1 Department of Pathology, Tokai University Oiso Hospital, 21-1 Gakyou, Oiso, Kanagawa 259-0198, Japan; 2 Department of Surgery, Tokai University Oiso Hospital, 21-1 Gakyou, Oiso, Kanagawa 259-0198, Japan

## Abstract

**Background:**

The case of adenocarcinoma with human chorionic gonadtropin (HCG), primary in the male gallbladder, is extremely rare. A Medline search has shown only a few similar cases reported.

**Methods:**

We herein describe a case of primary gallbladder adenocarcinoma associated by ectopic HCG positive tumor cells in a 79-year-old male.

**Results:**

Pathological examination showed a mixture of moderately and poorly differentiated adenocarcinoma with ectopic HCG and placental alkaline phosphatase (PlAP) in tumor cells, though the increase of serum or urinary HCG secretion was not confirmed. The literatures were also reviewed.

**Conclusions:**

A case of gallbladder cancer with ectopic HCG production is quite rare in the literature, though many similar cases in other site, especially in GI tract, are reported. Embryological consideration suggests the increased frequency of similar cases more than being thought now.

## Background

In gallbladder, human chorionic gonadotropin (HCG) related carcinoma is exceedingly rare in the literature. One case of adenocarcinoma with choriocarcinomatous element though negative by immunostain [1], two adenocarcinomas with choriocarcinomatous elements [2,3], one adenosquamous cell carcinoma [4], nine undifferentiated carcinomas [5] and another one extrauterine trophoblastic tumor (ETT) [6] have been reported. In other organs, most of the cases with HCG positive, non-gestational tumors have been reported as non-gestational choriocarcincoma in the gastrointestinal tract such as stomach [7-10], esophagus [11], colon [12,13] and jejunum [14]. Other sites such as brain [15], lung [16,17] and liver [18] have also been reported. In addition to the tumor with choriocarcinomatous elements, ectopic production of HCG by gastrointestinal tract tumors has also been reported by many authors either by using radioimmunoassay [19-22] or by immunohistochemical stain techniques [23-26].

Here we report a case of adenocarcinoma with ectopic HCG of the gallbladder. The literatures are also reviewed.

## Case report

A 79-y-old Japanese male was admitted to the Samukawa Hospital (Kanagawa, Japan) because of jaundice in June 2009. Computer tomography (CT) scan and magnetic resonance imaging (MRI) of the abdominal organs showed a mass lesion in the gallbladder, and the obstructive jaundice due to malignancy was suspected. He had no significant past history. Then he was admitted to the Tokai University Oiso Hospital (Kanagawa, Japan) for further examination and surgical treatment. After the additional examinations, clinical diagnosis of gallbladder cancer was made and cholecystectomy was performed at July 7, 2009. No tumorous lesion, suggestive of primary site, was identified in other organ including testis, mediastinum or gastrointestinal tract by CT scan, MRI or other examination.

For light microscopy, the specimen was fixed in 10% buffered formalin, and 4 mm-thick tissue slices were embedded in paraffin. Paraffin sections were stained with HE. Immunohistochemical detection of various markers in tumor was performed by the indirect immunoperoxidase method using antibodies (AE1/AE3; Ventana, HCG; Dako, βHCG; Dako, human placental lactogen (HPL); Dako, placental alkaline phosphatase (PlAP); Dako, chromogranin-A (CGA); Dako, CD56; Leika, Insulin; Dako, Glucagon; Dako, Gastrin; Dako, Prolactin; Dako, alfa-fetoprotein(AFP); Dako, Japan) [27,28].

## Pathological Findings

**Figure 1:**
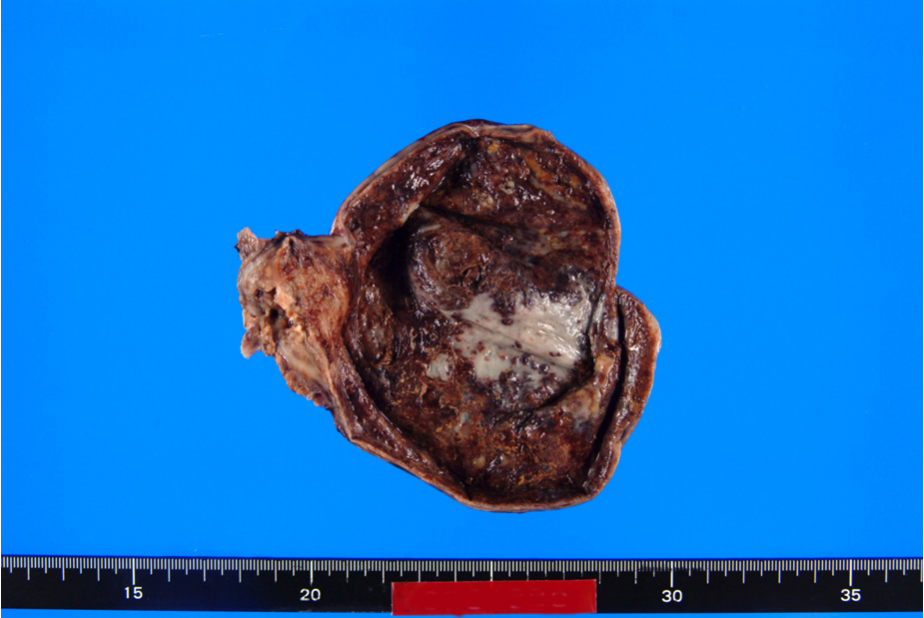
**The resected gallbladder with a large, ulcerative tumor mass measuring 4.5 cm × 3.5 × 2.5 cm with severe necrotic change**.

**Figure 2:**
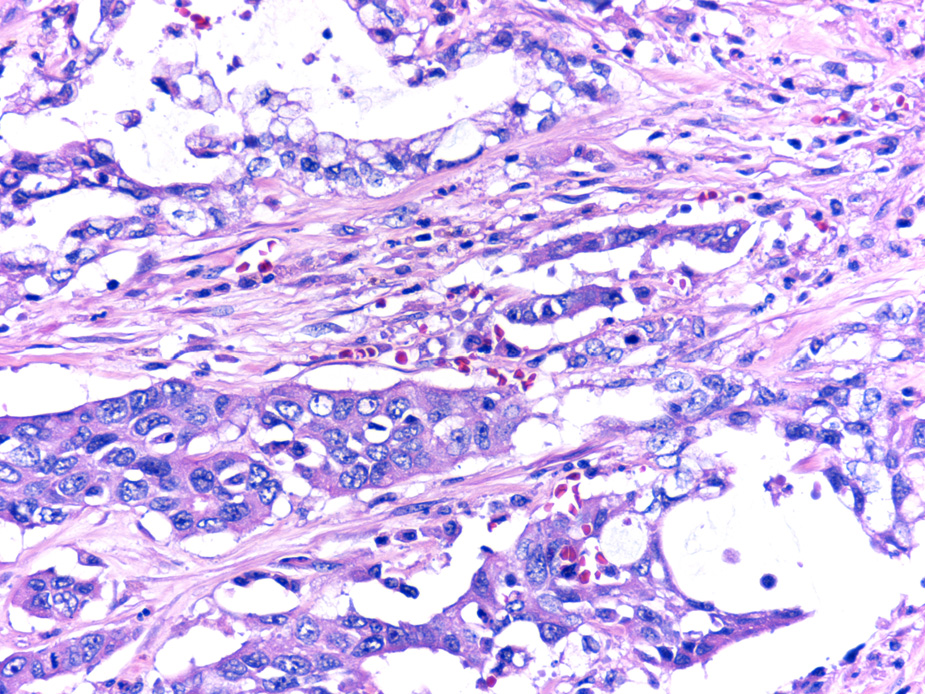
**Tumor composed of two elements, moderately differentiated tubular adenocarcinoma (upper one third) and rather poorly differentiated lesion with abortive tubular structures (lower two thirds)**.

Pathological examination of the resected gallbladder revealed a large, ulcerative tumor mass measuring 4.5 cm × 3.5 × 2.5 cm occupying more than two thirds of the gallbladder wall with necrosis (Fig. 1). Microscopic examinations showed two elements, moderately differentiated tubular adenocarcinoma and rather poorly differentiated lesion with abortive tubular structures (Fig. 2). The tumor invaded through the muscle wall but no extension through the serosa. The TNM stage was T2N0Mo. Residual gallbladder revealed moderate acute and chronic inflammatory cell infiltration and mild to moderate fibrosis in the wall, though neither the features of cholelithiasis nor porcelain gallbladder were evident.

**Figure 3:**
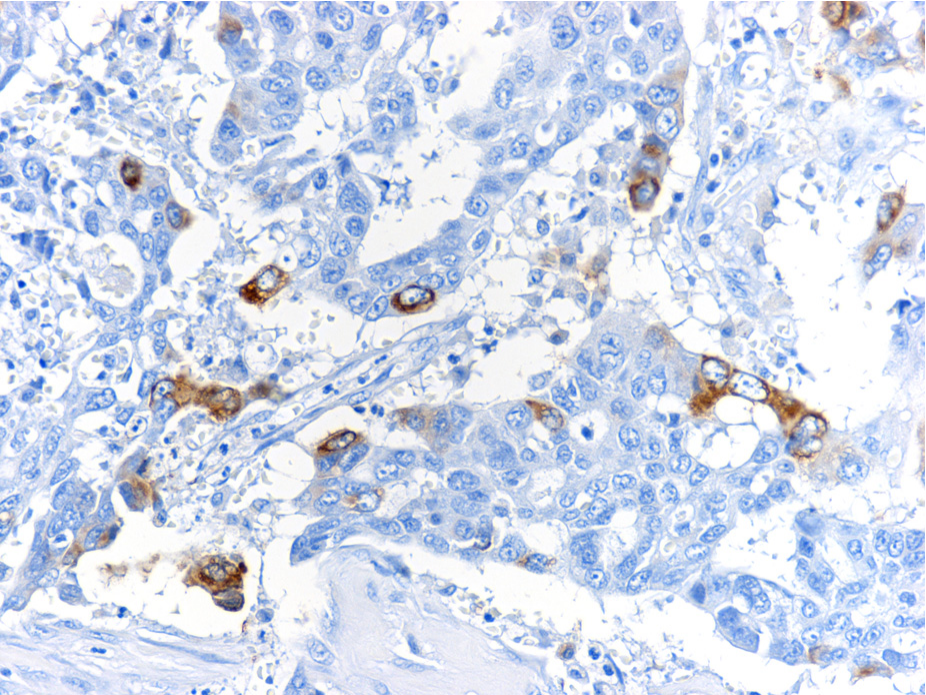
**Immunohistochemical staining for βHCG in the highly atypical tumor cells of poorly differentiated adenocarcinoma**.

**Figure 4:**
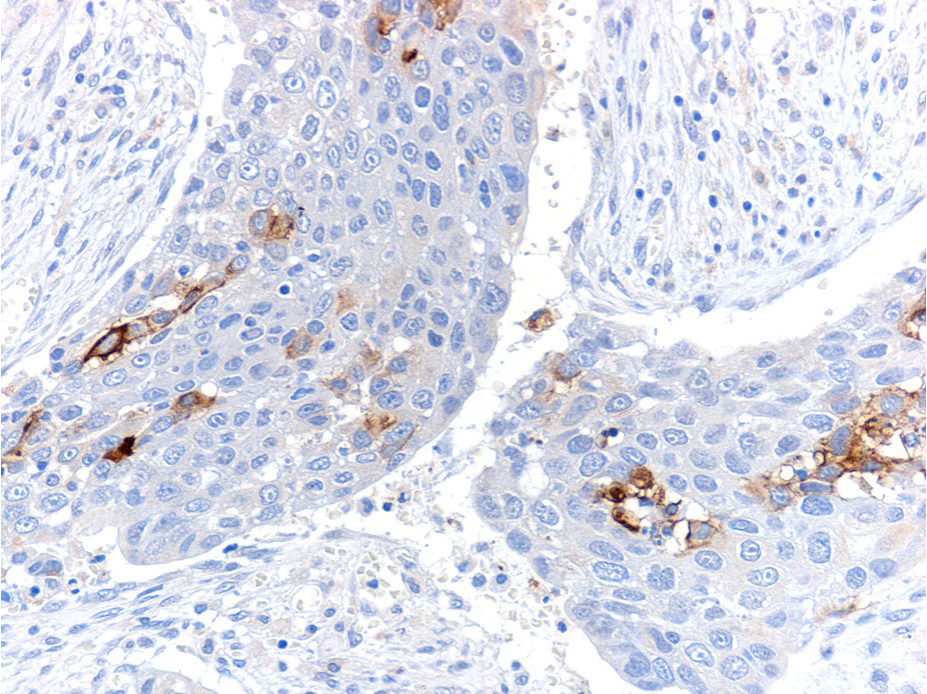
**Immunohistochemical staining for placental alkaline phosphatase (PlAP) in several tumor cells**.

Immunohistochemistry revealed positive staining for cytokeratin AE1/AE3 in all tumor cells, HCG and βHCG in the highly atypical tumor cells of poorly differentiated adenocarcinoma (Fig. 3) and for placental alkaline phosphatase (PlAP) in several tumor cells (Fig. 4). Other antigens including HPL, CGA, CD56, insulin, glucagon, gastrin, prolactin and AFP were negative.

Later than one month after the operation, serum HCG titer was measured for the first time and was normal, less than 1 mU/ml. Because of an old male patient with gallbladder tumor, preoperative evaluation of HCG was not performed.

Postoperative chemotheraty with anti-metabolic drug, Gemcitabine hydrochloride, was done and no apparent clinical evidence of recurrence has been obtained yet, more than 11 months after the operation.

## Discussion

Although quite rare, some cases of gallbladder carcinoma with HCG have been described in the literatures (Table 1). The histological diagnoses of these reports were as follow; choriocarcinoma [1-3], adenosquamous cell carcinoma [4], undifferentiated carcinoma with HCG [5], and extrauterine epithelioid trophoblastic tumour (ETT)[6].

**Table 1:** Clinicopathological Summary of Patients with Gallbladder Carcinoma and HCG

No	Age/sex	Cholelithiasis	Macroscopic type	Microscopic type	Immunostains	s/u HCG	Ref. No
1	63/F	+	Non-specific	AC + Choriocarcinoma	HCG (-)	ND	1

2	29/F	+	Protruded	AC + Choriocarcinoma	HCG (+), AFP (-), CEA (-)	Elevated	2

3	48/F	-	Protruded	AC + Choriocarcinoma	HCG (+)	Elevated	3

4	83/F	-	Protruded	ASC	HCG (+), AFP(-), CEA (+)	Elevated	4

5	68/F	-	Infiltrating	UC, pleomorphic type	HCG (+), VIM (+), CEA (+)	ND	5

6	65/F	+	Protruded	UC, pleomorphic type	HCG (+), Som (+), CEA (+)	ND	5

7	68/F	+	Protruded	UC, pleomorphic type	HCG (+), PP (+), Som (+), Gas (+), CEA (+)	ND	5

8	68/F	-	Infiltrating	UC, pleomorphic type	HCG (+), Gas (+), CEA (+)	ND	5

9	69/M	-	Protruded	UC, spindle cell or ps	HCG (+), Vim (+), Gas (+), CEA (+)	ND	5

10	61/M	-	Protruded	UC, spindle cell or ps	HCG (+), Vim (+), Som (+), Gas (+), CEA (+)	ND	5

11	66/M	-	Protruded	UC, spindle cell or ps	HCG (+), Vim (+), PP (+), CEA (+)	ND	5

12	61/F	+	Protruded	UC, spindle cell or ps	HCG (+), Vim (+), PP (+), Ser(+), Som (+), Gas (+), CEA (+)	ND	5

13	62/F	+	Infiltrating	UC, spindle cell or ps	HCG (+), Vim(+), Ser(+), Som(+), Gas (+), CEA (+)	ND	5

14	41/F	ND	ND	ETT	HCG (+), PlAP (+)	Elevated	6

15	79/M	-	Protruded	AC	HCG (+), PlAP (+)	NM	*

s/u:serum and/or urinary; HCG:human chorionic gonadotropin; AC:adenocarcinoma; ND:not described; AFP:alfa-fetoprotein; CEA:carcinoembryonic antigen; ASC:adenosquamous cell carcinoma; UC:undifferentiated carcinoma; Vim:vimentin; Som:somatostatin; PP:pancreatic polypeptide; Gas:gastrin; ps:pseudosarcomatous; Ser:serotonin; ETT: extrauterine trophoblastic tumor; PlAP:placental alkaline phosphatase; NM:not measured; *:our case.

Many cases of non-gestational choriocarcinoma have been reported in gastrointestinal tract or other organs [7-18], though only a few cases were observed in the gallbladder. The case reported as non-gestational, extra-uterine choriocarcinoma, primary in the gallbladder, was first described by Albores-Saavedra and coworkers [1]. The diagnosis of gallbladder adenocarcinoma with choriocarcinoma-like areas was only made by histological examination, though immunostains for the βHCG were negative and serum or urinary βHCG level was not described.

Abu-Farsakh and Fraire represented a second case of coexisting adenocarcinoma and choriocarcinoma of the gallbladder [2]. This may be the first case of such tumor documented by positive immunostaining for the βHCG and the increased levels of serum and urinaryβHCG, reported in the English language medical literature. Wang and coworkers reported another case of non-gestational choriocarcinoma in gallbladder [3]. This case also represented a mixture of adenocarcinoma and choriocarcinoma.

Fukuda and Ohnishi reported a case of gallbladder adenosquamous cell carcinoma with immunostain positive HCG accompanied by the remarkably increased serum and urinary HCG [4]. The primary lesion in the gallbladder was mainly composed of well differentiated adenocarcinoma and well differentiated squamous cell carcinoma, and metastatic tumor was composed of well to moderately differentiated adenocarcinoma. βHCG positive tumor cells were observed in the adenocarcinoma, especially in poorly differentiated areas.

As in these cases of gallbladder carcinoma, most of the reported cases of non-gestational choriocarcinoma in GI tract or other sites also revealed a coexistent adenocarcinoma component [7-17]. The origin of these choriocarcinomatous elements is still controversial. The various theories regarding pathogenesis include its development from ectopic germ cells or totipotent rests, teratoma, metaplastic differentiation, or metastasis from the intrauterine lesion.

Gastrointestinal tract tumors with ectopic production of HCG have been reported by many authors [19-26]. Fukayama et al reported the immunoreactive HCG in 51% of 124 cases of gastric carcinoma [23].

In our case, although neither apparent increase of serum or urinary HCG nor histologically typical choriocarcinoma element was confirmed, immunoreactive βHCG in adenocarcinoma cells was clearly identified especially in rather poorly differentiated lesion. These findings are consistent with the category, "adenocarcinoma with ectopic production of HCG" and the origin of HCG positive tumor cells in our case might represent a metaplastic differentiation or dedifferentiation.

Guo and coworkers described 21 cases of undifferentiated carcinoma of the gallbladder and βHCG positive tumor cells were observed in 9 cases, though serum or urinary level of βHCG was not described [5]. Cases were divided into three histological types; small cell type, pleomorphic cell type and spindle cell or pseudosarcomatous type. βHCG positive cancers were identified in four out of eight cases in pleomorphic cell type and all of five cases in spindle cell or pseudosarcomatous type tumors. In these nine cases with βHCG positive tumor cells, six cases were accompanied by gastrin, somatostatin and/or serotonin positive tumor cells. Authors suggested the neoplastic endocrine cells probably arising by divergent differentiation of the primitive neoplastic cells. These tumors may be categorized in "endocrine cell carcinoma".

Only one case of ETT in the gallbladder with markedly elevated HCG levels in urine and serum was reported in the literature [6]. ETT is a rare condition with approximately 50 reported cases in several organs and originated from cells of intermediate trophoblast. Immunohistochemical stain reveals positive reactions for epithelial markers, HCG and placental alkaline phosphatase, though quite different from our case in histological features.

As previously mentioned, the gastrointestinal tract tumors with ectopic production of HCG have been reported by many authors [19-26] and gallbladder is embryologically originates from the hepatic diverticulum, a ventral outgrowth from the intestine. If sensitive immunohistochemical studies were routinely done in all of gallbladder tumor, the case with immunoreactive HCG might be not a quite rare.

## Conclusions

A case of gallbladder cancer with ectopic HCG production is quite rare in the literature, though many similar cases in other site, especially in GI tract, are reported. Embryological consideration suggests the increased frequency of similar cases more than being thought now.

## Consent

Written informed consent was obtained from the patient for publication of this case report and accompanying images. A copy of the written consent is available for review by the Editor-in-Chief of this journal.

## Competing interests

The authors declare that they have no competing interests.

## Authors' contributions

SS drafted the manuscript and described the pathology component and took photographs, participated in writing the discussion, coordinated and edited the manuscript. MI edited the clinical part of the manuscript. MI, TF, EI and YO provided the clinical data and edited the clinical case presentation. SS reviewed the entire manuscript, participated in writing the discussion and the pathology component and edited the manuscript. All authors read and approved the final manuscript.
